# Symptom burden of people with progressive ataxia, and its wider impact on their friends and relatives: a cross-sectional study

**DOI:** 10.12688/amrcopenres.13036.1

**Published:** 2021-11-30

**Authors:** Anja Lowit, Julie Greenfield, Emily Cutting, Ruby Wallis, Marios Hadjivassiliou

**Affiliations:** 1 School of Psychological Sciences and Health, University of Strathclyde, Glasgow, G1 1QE, UK; 2 Ataxia UK, London, N6 5JW, UK; 3 Academic Department of Neurosciences, Sheffield Teaching Hospitals NHS Trust, Sheffield, S10 2JF, UK

**Keywords:** Ataxia, Symptoms, Quality of life, Activities of daily living, Carers, Symptom burden

## Abstract

**Background:**

Progressive ataxias are complex disorders that result in a wide variety of symptoms. Whilst we currently have a relatively good understanding of the symptom patterns associated with the various types of ataxia, and how these diseases progress over time, their impact on the person with ataxia is less well understood. In addition, little is known about how carers, friends and families are affected by them. This paper aims to provide preliminary information on the presence and impact of medical symptoms and day-to-day challenges on people with ataxia and their friends and relatives.

**Method:**

Data were extracted from a survey by Ataxia UK for their members. The views of 366 people with ataxia and 52 friends and relatives are reported. Data were analysed for the entire groups, as well as for the three most common ataxia types represented in the sample, Friedreich’s ataxia, inherited ataxia (excluding Friedreich’s ataxia), and cerebellar ataxia of unknown cause.

**Results:**

The survey confirmed the symptom patterns described in previous research, but further showed that the impact of these symptoms can vary across ataxia populations. Similar findings were observed for day-to-day challenges. Friends and relatives experienced similar challenges to people with ataxia, indicating that support provided has to consider those supporting people with ataxia as well as the patient. Respondents also highlighted limitations in terms of accessing support services, and not all services were able to cater fully to their specific needs.

**Conclusion:**

This study begins to provide information that can be used in further research to explore the needs of people with ataxia and their carers, friends, and relatives. Such research will support treatment trial design, ensuring patients’ needs are considered, help to tailor support services to their needs, and ensure health care professionals have the necessary skills to fully address them.

## Plain language summary

Ataxias are movement disorders that result in poor coordination and can include a variety of other difficulties, including speech difficulties, heart conditions, and problems with eyesight. Every type of ataxia results in a different pattern of symptoms, and they also differ in the age at which symptoms first appear. In progressive ataxias, symptoms become more severe over time.

The medical symptoms often impact daily living, such as moving about, eating and drinking, socialising, or the ability to work. They also affect those who live with or look after the person with ataxia. It is therefore important to collect people’s views about how the symptoms of their condition affect their daily lives, and what kind of support helps to alleviate these.

This paper reports the results of a survey of people with ataxia and their friends and relatives to explore these questions. It was highlighted that people with ataxia deal with a wide variety of medical symptoms and that these have important effects on their lives. It also showed that those caring for them are similarly affected and also need support.

The study has highlighted several areas where more research needs to be conducted to provide effective support for people with ataxia and their friends and relatives, and to inform the design of treatment trials to ensure the needs of patients are being addressed.

## Introduction

Ataxia refers to a constellation of symptoms and signs resulting from damage to the cerebellum. These include loss of co-ordination (affecting limbs and gait), clumsiness, tendency to fall, slurred speech, and sometimes visual disturbance referred to as oscillopsia (jumpy and blurred vision). Ataxia can also result in cognitive deficits referred to as the cerebellar cognitive affective syndrome. The causes of ataxia are diverse but can broadly be divided into genetic, degenerative and immune-mediated.

The prevalence of different genetic ataxias varies across different geographical regions. The most common genetic ataxia in the UK is thought to be Friedreich’s ataxia (FRDA) ^1^, an autosomal recessive, early onset (under 20 years of age) ataxia that frequently results in severe disability with wheel-chair dependency and limited lifespan. It is also associated with cardiac (cardiomyopathy), spinal (scoliosis) and endocrine (diabetes) dysfunction, making it a truly multi-system disorder. Spinocerebellar ataxia type 6 (SCA6) is the most common autosomal dominant ataxia in the UK ^2^. SCA6 presents later on in life (usually over 50 years of age) and unlike FRDA it is a pure ataxia without associated extracerebellar features. Patients with SCA6 can also be significantly disabled by their ataxia but usually have a normal lifespan. Some genetic ataxias may be less disabling. For example, episodic ataxia type 2 (EA2), a relatively common autosomal dominant ataxia, can be associated with slow progression and episodes of ataxia that are self-limiting and often can be prevented by the use of medication ^3^. Cerebellar variant of multi-system atrophy (MSA-C) is the best example of a degenerative ataxia that usually presents at 50–60 years of age, with a combination of slurred speech, gait ataxia, and autonomic dysfunction characterised by urinary and postural symptoms ^4^. The progression is fast with severe disability and on average survival from symptom onset to death is about 10 years. There is significant variability amongst patients with immune mediated ataxias, some following a rapidly progressive course (paraneoplastic cerebellar degeneration), whilst others progress slowly over many years (gluten ataxia) ^5^.

Not all patients with ataxia will have all the ataxia symptoms, the same degree of severity or the same rate of progression. Indeed, the variability of the above can be a useful tool in the diagnostic workup for the cause of the ataxia: acute and rapidly (days to weeks) progressive ataxia is a characteristic of paraneoplastic cerebellar degeneration ^5^. The early presence of significant dysarthria and the presence of autonomic dysfunction are features of MSA-C. Early onset disease usually has a genetic explanation ^1^.

Ataxias in general are associated with significant disability and thus high impact on the quality of life of both the people living with ataxia, as well as their friends and relatives who are often also their carers.

Many studies looking into “health related quality of life” (HRQOL) have applied standard generic tools such as the SF36, EQ-5D VAS or Activities of Daily Living (ADL). These include studies on specific diseases both at single points in time, e.g. children and adults with FRDA ^6– 10^ and patients with autosomal dominant spinocerebellar ataxias (SCA) ^11^, as well as over the course of several years to capture progression of the disorder, again involving patients with FRDA ^12, 13^ and SCAs ^14^. These studies have established reductions in HRQOL, the degree of which can often be linked to the severity of the disease. The awareness that HRQOL is an important aspect to consider in measuring treatment outcomes has led to the development of a number of scales that go beyond capturing purely clinical (mainly motor) symptoms but also investigate the impact of these on functional activities of daily living, social interactions, mental health and wellbeing. Such scales include the FRDA Impact Scale (FAIS ^15^), with a pool of 126 items that capture eight areas: speech, upper limb functioning, lower limb functioning, body movement, complex tasks, isolation, mood, and self-perceptions. More recently, a 114 item Patient Reported Outcome Measure of Ataxia (PROM-Ataxia) ^16^ has been developed from feedback from participants with a range of ataxias and covers a variety of physical symptoms, including upper and lower limb functioning, speech, vision, bowel movements, etc, as well as ADL and mental components (divided into mental health and cognitive functioning). In addition, a Disease Consequence Model for people with FRDA has been developed ^17^, which establishes the physical symptoms (classified by systems such as nervous system, multi-system), the impact they have on performance, and which daily activities they impact on, e.g. nervous system, voice/speech, slurred speech, difficulty being understood, and impact on personal, domestic and community daily activities. The model can form the basis of discussion with patients on how specifically their symptoms limit their lives and thus impact on quality of life. Finally, research is currently ongoing by Zizzi and colleagues to develop a PROM for FRDA, called Friedreich’s Ataxia Health Index (FA-HI), which will be a measure of the disease burden experienced by this patient group. Data published to date focuses on interviews conducted to explore which symptoms and other issues had the greatest impact on their lives ^18^. The research focused on both adults (18+ years) and children. The latter were either interviewed directly (11-17 years of age) or caregivers were questioned on their behalf in the case of children below 10 years of age. Answers were grouped into 22 symptomatic themes. They report that the top ten most frequently mentioned symptoms that impact on the lives of adults with FRDA were: 1) limitations with mobility or walking; 2) emotional issues; 3) social role limitations; 4) activity limitations; 5) hand and finger weakness; 6) fatigue; 7) social role dissatisfaction; 8) pain; 9) communication difficulties; and 10) choking or swallowing issues. Some differences between the age groups emerged, which were to some degree related to symptom onset, e.g. communication and swallowing issues were not as prevalent. On the other hand, less children reported that emotional issues impacted them strongly, whereas pain featured more prominently in their interviews. Caregiver reports representing the youngest age group showed a different picture again, with emotional issues nearly as prevalent as mobility limitations. In addition, social role limitations were the most frequently mentioned symptom in this group. Further research is required to establish to what degree these differences are due to actual age issues or differences in perception across children with FRDA and their carers.

These scales and models form a valuable resource for clinicians and researchers to identify the nature and severity of medical and non-medical symptoms present in people with ataxia that can serve as useful tools in their management. In addition, they make important contributions to disease specific outcome measures, used both in clinics and as tools to evaluate the effectiveness of interventions in clinical trials. However, whilst they provide an overview of the most common challenges, the methodologies applied in most of these studies, with the exception of 18 (which focuses only on FRDA) do not allow extrapolation of which of these symptoms tend to have the highest impact for the patient population as a whole. In addition, the above scales cover a limited range of ataxia types, and no studies to date have explored whether different ataxia populations are impacted in similar ways. Such information is essential for future service planning and resource allocation to ensure effective, person-centred clinical management, and are also important in the assessment of the impact of a new treatment on patients.

A further significant knowledge gap concerns the carers, family and friends of people with ataxia. Support provided to the family of those with progressive neurological conditions is important to provide general every day support and also to facilitate effective intervention ^19^. Yet, whilst a number of studies have focused on carers of patients with slow and fast progressing neurological disorders such as Parkinson’s disease, multiple sclerosis, dementia as well as motor neuron disease, Huntington’s disease and multiple system atrophy ^20– 23^, no reports exist, to our knowledge, that investigate the challenges faced by carers, family and friends of people with ataxia and thus how to support them effectively.

In 2016, Ataxia UK surveyed their members to aim to better understand their needs and the impact of ataxia in order to inform research priorities both for Ataxia UK and the general ataxia community. The survey contains valuable information to address some of the knowledge gaps identified in the current literature. This paper contributes to the ongoing exploration of these areas by reporting relevant sections of the survey, focussing on:

1. What are the medical/health symptoms experienced by people with ataxia and what is their impact?
2. What are the difficulties experienced in day-to-day living by people with ataxia and what is their impact?
3. What are the difficulties experienced by friends and relatives of someone with ataxia and what is their impact?
4. What support services do people with ataxia and their friends and relatives access, and what is their experience of these?

## Methods

### Participants

The data were retrospectively extracted from relevant sections of a survey distributed to people with ataxia. The survey had been designed by Ataxia UK, which is the national charity that supports patients with ataxia in the UK.

Data were collected by means of an anonymous, cross-sectional survey with a purposive and snowball sampling strategy. It was distributed to the entire membership of Ataxia UK, which consisted of approximately 4500 households in July 2016, who were invited to also pass the survey information on to others in their social circle to increase the response rate.

The survey was designed for anyone affected by ataxia either through having the condition or being close to someone who has ataxia (henceforth referred to friends or relatives although not all respondents had this relationship, see respondent profile below). There were no restrictions in terms of underlying pathology of the ataxia in recognition of the wide membership range the charity caters for.

Ataxia UK’s Internal Ethics Committee reviewed and approved the publication of the survey results in July 2021.

### Survey content

The survey took the form of a questionnaire. This was mailed to the membership together with a paid envelope to allow them to return the completed form by post. Alternatively, respondents were able to complete the questionnaire online using the CloudSurvey platform.

The questionnaire consisted of 22 items, covering (1) respondents’ demographics and medical background, (2) the effects and impact of ataxia, (3) sources of help and their effectiveness, and (4) the role and services of Ataxia UK. The relevant questions analysed in this paper can be found in the *Extended data* ^24^.

People with ataxia answered all sections, those identified as friends or relatives did not complete sections on medical symptoms and some day-to-day challenges that would not apply to them.

The questions relating to the medical symptoms as well as day-to-day challenges were sourced from previous Ataxia UK surveys, anecdotal reports of people with ataxia that Ataxia UK had gained from their Helpline and close contact with people with ataxia and from the literature ^3, 25, 26^. The items closely align with previous and subsequently published data ^15– 18^.

### Data analysis

Data were analysed quantitively through descriptive statistics using Microsoft Excel©. Comparative statistical methods were not deemed appropriate for this sample as the survey had not been designed to identify group differences or relationships between different variables.

## Results

### Respondent profile

We received 426 responses to the survey, representing a response rate of 9% of the number of questionnaires distributed (n=4500). Some responses lacked essential demographic information and were thus removed from analysis, resulting in a total of 418 questionnaires. Most respondents were white (96%). Three people described themselves as mixed race, six as Asian or Asian British and four as Black or Black British. A total of 12% (n=52) of all respondents were friends or relatives of someone with ataxia, and 88% (n=366) had ataxia themselves. With an estimated population of around 10,000 people with ataxia in the UK, this corresponds to approximately 3.5% of the ataxia population.

**Figure 1.**
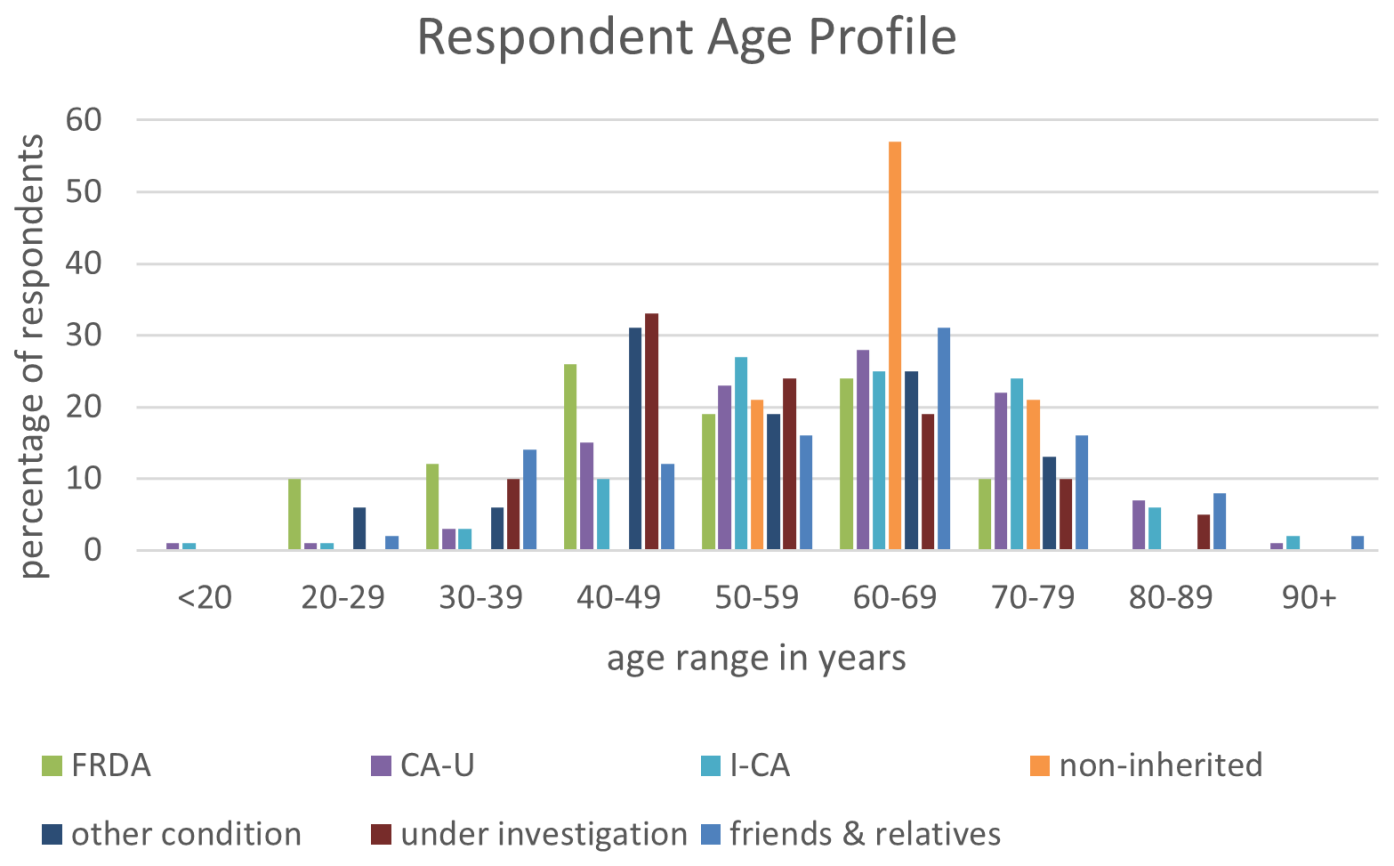
Respondent age profile. Abbreviations: *CA-U = cerebellar ataxia of unknown cause, I-CA = inherited ataxia (excluding FRDA), FRDA = Friedreich’s ataxia. “Other condition” combines data for all other groups listed in Table 1*.

Table 1 provides information about the distribution of ataxia types represented in this sample. Of those respondents with confirmed diagnoses, just over a third had cerebellar ataxia of an unknown cause (CA-U), followed by those with inherited ataxias excluding FRDA (I-CA). Sixty of the 98 respondents with I-CA provided details of their diagnosis, showing a wide range of ataxia types, with the largest groups representing those living with spinocerebellar ataxia type 6 (SCA6), followed by SCA2 and episodic ataxia type 2 ( Table 2). The frequency of the different SCAs is representative of the prevalence in the UK population. Individuals with FRDA formed the next largest group, with the remaining respondents distributed relatively equally across the categories of non-inherited ataxias, ataxia associated with other conditions such as brain tumour, those still under investigation for ataxia type, and unspecified “other” problems. There was wide variation in the age range of respondents, with the majority being between 50 and 80 years old ( Figure 1). Of the four groups with confirmed diagnoses, the FRDA respondents had the youngest age profile, whereas those with other types of ataxia tended to be older than 50 years. The other two categories with younger respondents were those with other health conditions, and those still waiting for a diagnosis. The age profile of friends and relatives followed a similar pattern, peaking around 50–80 years.

**Table 1.** Respondent categories and distribution.

	All PwA	Friends or relatives	CA-U	I-CA	FRDA	Non-inherited ataxia	Other condition	Under investigation	Other
**n**	366	52	136	98	42	15	17	23	35
**In relation to all PwA**			37%	27%	4%	5%	11%	6%	10%

*Abbreviations: n = number, PwA = person with ataxia, CA-U = cerebellar ataxia of unknown cause, I-CA = inherited ataxia (excluding FRDA), FRDA = Friedreich’s ataxia*

**Table 2.** People with inherited cerebellar ataxia diagnostic profile.

I-CA diagnosis	Percentage	I-CA diagnosis	Percentage
**SCA1**	7	**SCA 14**	2
**SCA 2**	13	**SCA 20**	2
**SCA 3**	5	**ARSACS**	2
**SCA 5**	2	**AOA2**	3
**SCA 6**	45	**EA1**	2
**SCA 7**	3	**EA2**	8
**SCA 8**	3	**SPG7**	2
**SCA 11**	2		

*Abbreviations: n = number, SCA = Spinocerebellar Ataxia, ARSACS = Spastic Ataxia Charlevoix-Saguenay Type, EA = Episodic Ataxia , SPG7 = Spastic paraplegia Type 7. Note that the percentage of I-CA diagnosis relates to the total number of diagnoses provided (n=60) rather than all PwA with an I-CA diagnosis (n=98).*

The paper will report responses for all people with ataxia, and in addition, provide information on some of the subgroups of respondent to inform on potential differences in symptoms and impact across ataxia types. This subgroup analysis will focus on CA-U, FRDA and I-CA given the predominance of these conditions in the respondent sample.

The remaining 52 respondents that did not have ataxia themselves indicated that three different categories applied to them: ‘people who lived with a relative with ataxia’ (n=26), ‘friends or relatives of someone with ataxia’ (n=37) and the category 'Other’ (n=15). As evident by the higher total, some respondents ticked more than one category, in most cases the first two choices, i.e. those who were a friend or relative of and who actually lived with a person with ataxia. In addition, 7% of respondents with ataxia were also carers to someone else with ataxia. Of the 15 responses in “Other” category, 9 indicated that they had a family member with ataxia, although in some cases deceased. The remaining six people included two carers, three people who expressed an interest in ataxia, and in some cases were involved in Ataxia UK activities. It is therefore reasonable to assume that most respondents had a close relationship to people with ataxia, and around 50% lived with and cared for them.

### Medical symptoms experienced by people with ataxia

Respondents were asked to indicate which health problems they had experienced in the past, by ticking all that applied from a list of 40 items, plus a free text option, covering coordination/balance/mobility, posture, incontinence, involuntary movements, pain, and other health problems. The Top 10 issues reported across all participants included, in order of magnitude, balance, walking, clumsiness, falling, speech problems, standing, dexterity, fatigue, incontinence, and coughing/choking ( Table 3). These affected more than 60% of respondents in each case. More detailed investigation of the CA-U, I-CA and FRDA groups showed some variation though. For example, a considerably higher percentage of people with FRDA reported issues with dexterity, bladder incontinence, coughing/choking, involuntary muscle spasms, back pain, hypophonia, poor circulation, cold extremities, ankle inversion, saliva problems, scoliosis (without spinal rod) and bowel incontinence. This did not apply across all symptoms though, e.g. double vision was not a prominent feature in FRDA compared to the other two conditions. Respondents with I-CA reported a higher incidence of nystagmus, headaches and heart problems as well as weakness, leg/arm pain and double vision. Responses from people with CA-U stood out by a considerably lower number of speech problems reported, and they generally had a lower number of respondents reporting symptoms compared to the other two groups with no indication that particular symptoms were more prevalent in this population.

**Table 3.** Percentage of people reporting medical symptoms (in order of frequency reported across all people with ataxia).

Health problem	All PwA (n=366)	CA-U (n=136)	I-CA (n=98)	FRDA (n=42)
**Balance problem/unsteadiness**	97	96	99	95
**Walking**	90	90	91	90
**Clumsiness**	86	85	91	95
**Falling**	80	83	80	83
**Slurred speech/dysarthria**	74	68	82	88
**Standing**	73	75	72	76
**Using hands/dexterity**	70	69	73	86
**Tiredness/fatigue/lack of stamina**	68	64	72	74
**Bladder incontinence**	60	34	37	52
**Coughing/choking**	47	57	65	79
**Weakness**	47	35	57	48
**Dizziness**	45	46	51	57
**Swallowing**	39	43	46	50
**Restless leg**	38	34	42	48
**Involuntary muscle spasms**	38	29	44	67
**Back pain**	38	29	36	57
**Leg or arm pain**	37	30	44	38
**Poor circulation**	34	23	39	67
**Unable to speak loudly enough**	32	29	35	45
**Double vision**	32	24	48	5
**Cold extremities**	31	31	31	50
**Nystagmus**	29	21	39	26
**Involuntary tremor**	28	20	34	29
**Drooling**	27	24	31	24
**Headache**	27	19	38	24
**Overweight**	23	18	31	26
**Hearing**	23	22	23	21
**Other mobility problems**	18	13	16	26
**Other pain**	17	11	15	21
**Inversion of the ankles**	16	10	13	45
**Other posture problems**	15	14	13	19
**Heart**	13	7	16	5
**Other saliva/mucus problem**	13	8	10	36
**Other**	13	12	13	5
**Dystonia**	11	7	11	17
**Scoliosis without spinal rod**	9	4	5	31
**Bowel incontinence**	9	5	10	19
**Partial loss of sight**	8	5	9	5
**Diabetes**	8	9	8	10
**Severe visual impairment**	6	4	6	7
**Scoliosis with spinal rod**	2	1	2	2

*Abbreviations: PwA = people with ataxia, CA-U = cerebellar ataxia of unknown cause, I-CA = inherited ataxia (excluding FRDA), FRDA = Friedreich’s ataxia*

We also investigated how many health symptoms each respondent experienced. Out of the 41 possible options, people with ataxia reported a minimum of 7 and a maximum of 39 issues, with a median of 16. This did not differ noticeably across the groups, i.e. the I-CA group had a median of 17 with a range of 7 to 39, and the FRDA group a median of 18 with a range of 7 to 32. As suggested above, only the CA-U group was slightly less affected with a median of 14 and a range of 7 to 29. Interestingly, all groups reported a minimum of 7 symptoms, reflecting the multifaceted nature of each type of ataxia.

### The impact of health problems experienced by people with ataxia

The next question in the survey focused on which of the reported health problems impacted most on the lives of the respondents with ataxia, ranking them in order of the three most impactful symptoms. These data are presented in relation to how many respondents had reported the problem rather than the total number of respondents.

**Figure 2.**
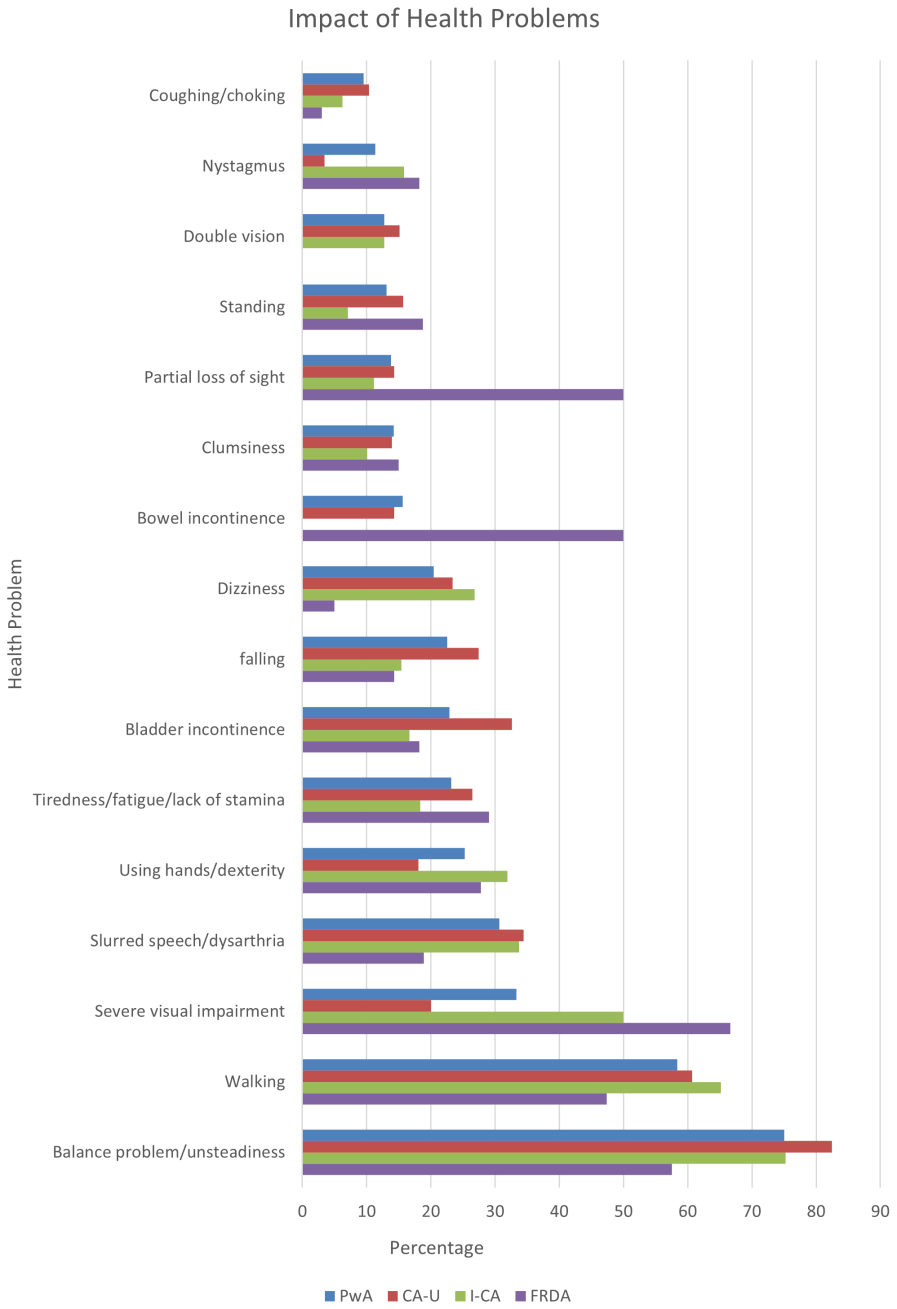
Health problems with the first, second and third highest ranked impact as a function of respondents reporting this symptom. Symptoms reported as impactful by less than 10% of all people with ataxia are not represented. Abbreviations: PwA = person with ataxia, *CA-U = cerebellar ataxia of unknown cause, I-CA = inherited ataxia (excluding FRDA), FRDA = Friedreich’s ataxia*

Considering only the symptoms that were scored as having the highest impact (first rank), balance problems were highlighted by 44% of those experiencing this issue, followed by walking difficulties which were the most impactful symptom for 25%. The other symptoms were said to have the highest impact in 14% or less of respondents. When the data for the three most highly ranked problems are pooled, balance and walking problems still feature as the two most impactful symptoms. In addition, speech problems (dysarthria) and severe visual impairments impacted on more than a third of respondents, and dexterity, fatigue, bladder incontinence, falling and dizziness were highly limiting factors for more than 20% of respondents ( Figure 2). The remaining symptoms were listed as having the highest impact by less than 20% of respondents reporting these issues.

Figure 2 also demonstrates that a high prevalence of a symptom ( Table 2) does not necessarily equate to its impact. Whilst walking and balance are both most widespread and most impactful across the respondents, other problems could show a different pattern. For example, clumsiness and falling were relatively common symptoms, experienced by around 80% of people with ataxia ( Table 2), yet less than 20% ranked them as one of their three most impactful symptoms ( Figure 2).

In addition, some variation was noticeable across the different types of ataxia. Whilst balance and walking problems ranked as the two most impactful problems for the overall PwA group, as well as those with CA-U and I-CA, these problems were an issue in a noticeably lower percentage of people with FRDA. People with FRDA also indicated less impact from speech disturbances and dizziness than these groups. On the other hand, they reported considerably higher impact from severe visual impairment or partial loss of sight, as well as bowel incontinence. The only other noticeable group differences relate to people with CA-U compared to the FRDA and I-CA groups in relation to falling and bladder incontinence, which had a higher impact in this population.

### Day-to-day challenges

***Day-to-day challenges experienced by people with ataxia.*** The next two sections of the survey asked about the types of challenges people with ataxia and their friends and relatives experience on a day-to-day basis. These were divided into those specifically affecting people with ataxia, and those that could affect both groups of respondents.

**Figure 3.**
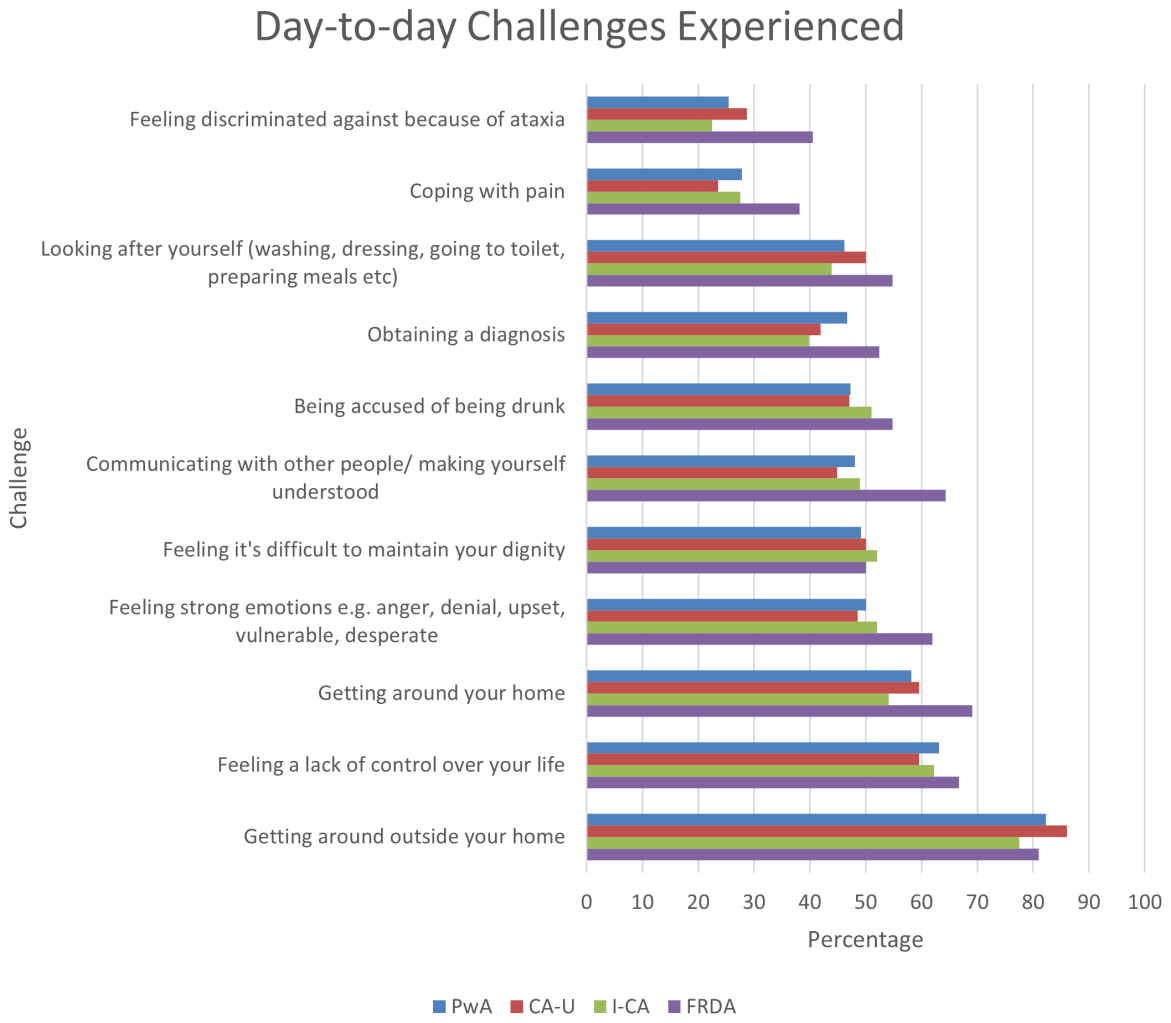
Day to day challenges experienced by people with ataxia. Abbreviations: PwA = person with ataxia, *CA-U = cerebellar ataxia of unknown cause, I-CA = inherited ataxia (excluding FRDA), FRDA = Friedreich’s ataxia*

In relation to the questions answered by people with ataxia only, Figure 3 shows that getting around outside the home is the most common challenge experienced across all types of ataxia. This is consistent with the health problems reported in Table 3 and Figure 2 (balance problems/unsteadiness and walking). Feeling a lack of control, and problems with mobility inside the home were furthermore experienced by over 50% of respondents with ataxia. At the lower end coping with pain and feeling discriminated against were less common with under a third reporting these as challenges they encountered. Generally, more people with FRDA indicated that they experienced the given problems, suggesting a broader spectrum of challenges within this group.

**Figure 4.**
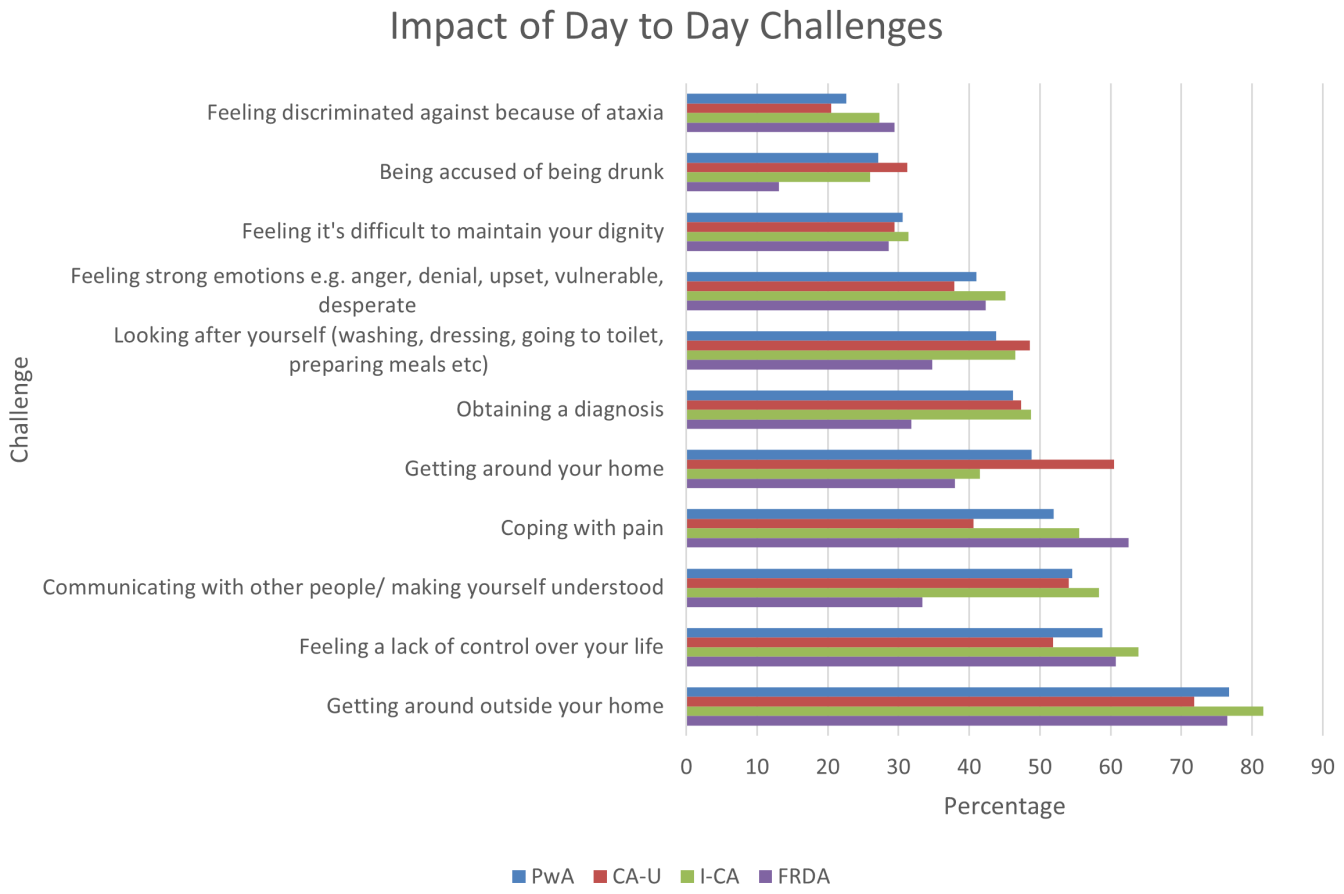
Day to day challenges with the first, second and third highest ranked impact as a function of respondents reporting this challenge. Abbreviations: PwA = person with ataxia, *CA-U = cerebellar ataxia of unknown cause, I-CA = inherited ataxia (excluding FRDA), FRDA = Friedreich’s ataxia*

***The impact of day-to-day challenges experienced by people with ataxia.*** Respondents were again asked to rank these day-to-day challenges based on the impact it has on their life (first, second and third greatest impact). Figure 4 shows that the most common challenge, mobility outside the home, also had the greatest impact. A feeling of lack of control had the second highest impact, followed closely by communication problems and coping with pain in respect to all people with ataxia. The remaining challenges impacted most on less than 50% of all respondents, however, there were again differences across the ataxia types. Interestingly, although people with FRDA had signalled a high prevalence of speech problems, they reported being the least affected by communication difficulties compared to the other two groups. On the other hand, they were the most impacted by pain. People with CA-U showed the highest impact in relation to getting around in the house, whereas mobility outside the home was more in line with the other groups.

### Wider challenges experienced by people with ataxia and their friends and relatives

The next section of the survey looked at challenges experienced by all respondents, i.e. people with ataxia as well as their friends and relatives. As indicated earlier, a small percentage of PwA had reported that they were also related to someone else with ataxia. Based on the assumption that they would mostly rate the challenges with respect to their own difficulties their data were included in the PwA group and excluded from the friends and relatives group.

**Figure 5.**
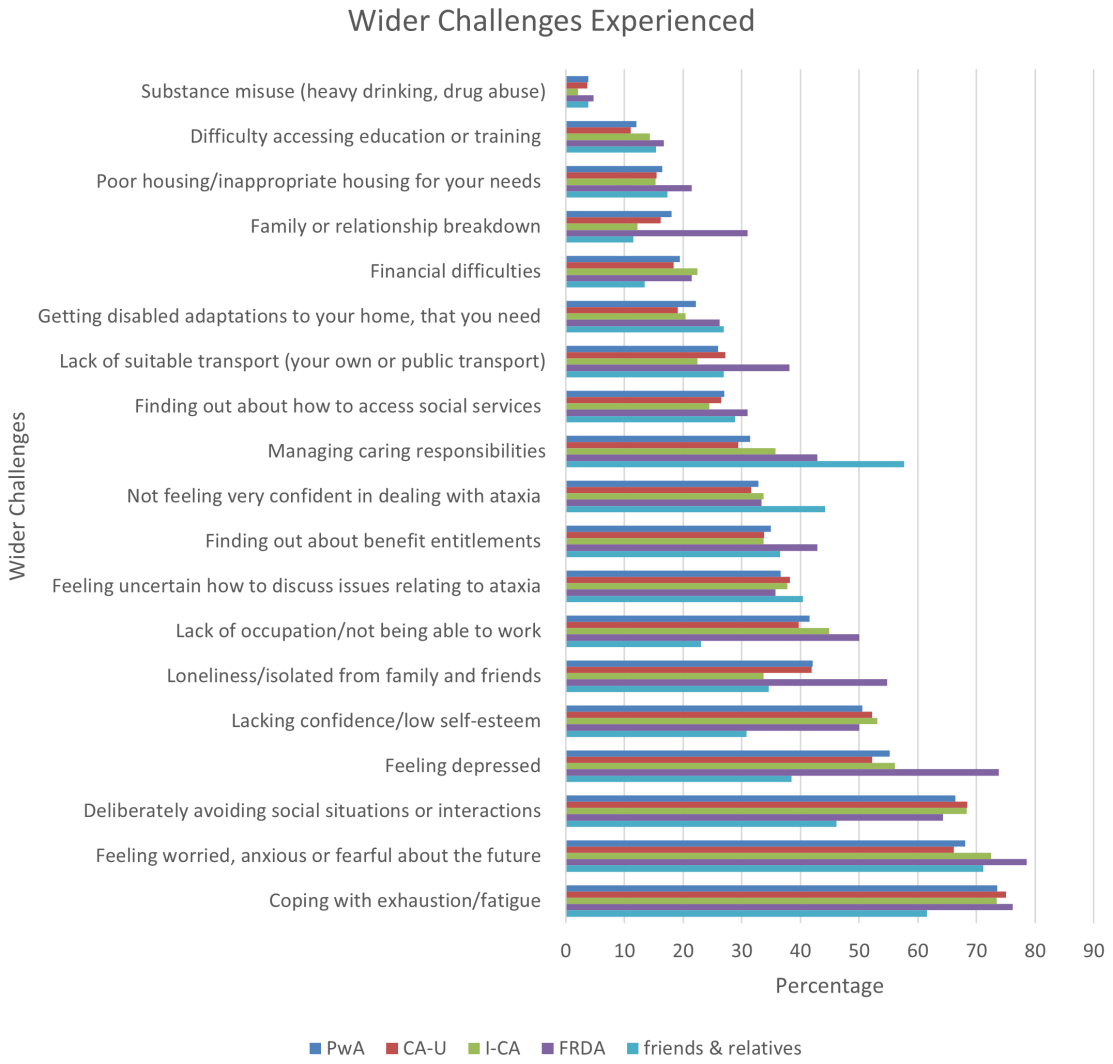
Wider challenges experienced by people with ataxia and their friends and relatives. Abbreviations: PwA = person with ataxia, *CA-U = cerebellar ataxia of unknown cause, I-CA = inherited ataxia (excluding FRDA), FRDA = Friedreich’s ataxia*

Figure 5 shows that the most common challenge for both PwA and their friends and relatives was coping with exhaustion or fatigue and high levels of anxiety or worry about the future. Whilst fatigue was a more prominent challenge for the PwA, about two thirds of friends/relatives also reported this as an issue for themselves. There was further evidence of greater presence of challenges in the PwA group in relation to psychosocial factors such as depression, loneliness, low self-esteem and reduced social participation, but again, at least one third of friends/relatives were equally affected by these issues. PwA also found not being able to work a challenge, but this did not feature too prominently in the friends and relatives group. Issues raised more frequently by the latter understandably related to managing caring responsibilities and lack of confidence in dealing with ataxia. Challenges relating to economical or practical issues such as financial difficulties, access to housing or appropriate transport were experienced by less than a third of respondents with no particular differences evident across the groups.

In relation to ataxia subgroups, Figure 5 shows that people with FRDA again showed some of the highest prevalence of challenges experienced compared to the other two groups. 

### The impact of challenges experienced by people with ataxia and their friends and relatives

**Figure 6.**
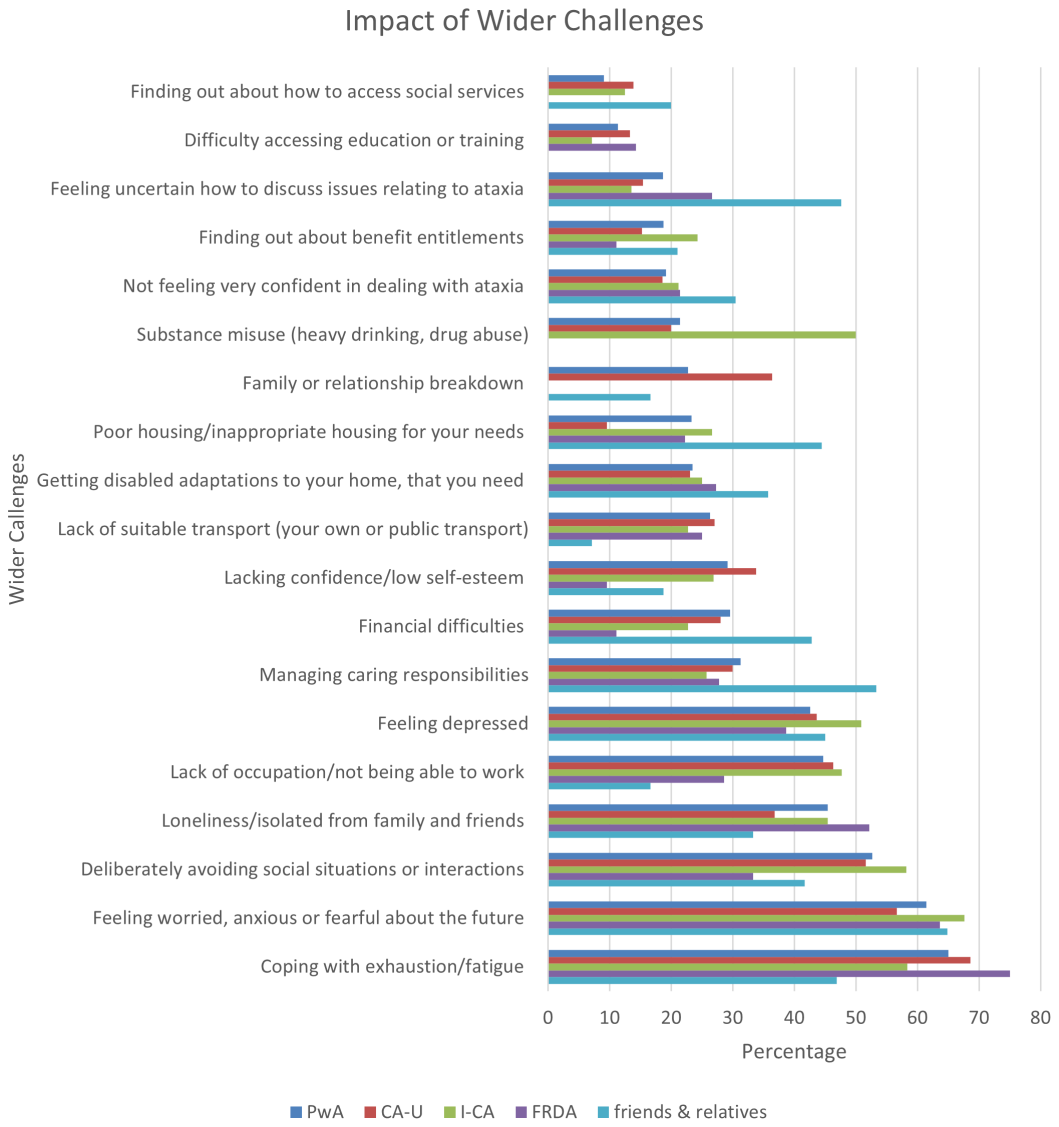
Wider challenges with the first, second and third highest ranked impact as a function of respondents reporting this challenge. Abbreviations: PwA = person with ataxia, *CA-U = cerebellar ataxia of unknown cause, I-CA = inherited ataxia (excluding FRDA), FRDA = Friedreich’s ataxia*

Respondents were again asked to rate the most impactful challenges. Although the question asked for the four most impactful issues, only data for the first three are reported here for consistency with previous sections. The cumulative figures for the most prominent challenges experienced by people with ataxia and their friends and relatives indicate that the impact of these challenges is very much in line with the frequency these have been reported, i.e. coping with fatigue, being worried about the future and avoiding social interactions feature as the three most prominent items in both Figure 5 and Figure 6. Similarly, some categories are more prevalent in one group than the other, e.g. PwA were more affected by not being able to work and a lack of confidence, whereas friends and relatives found it more difficult to deal with their caring responsibilities and having the confidence to deal with and discuss issues related to ataxia. The various subgroups of ataxia appeared to be similarly impacted by the challenges with the exception of the respondents with FRDA who reported less impact of social issues such as avoiding social situations, lack of confidence, and family/relationship breakdowns, as well as financial challenges, i.e. lack of occupation and financial difficulties. On the other hand, being able to discuss issues relating to ataxia had a slightly higher impact.

### Support sought by people with ataxia

***Support from health and social care and medical advice.*** In this section of the survey, people were asked which services they had used for help or advice related to ataxia, and to indicate any services they would like to use but cannot access. This section only focuses on the respondents with ataxia. Table 4 shows the services PwA used or would have liked to use if the service had been available for them. The final column represents the presumed unmet need of each service, expressed as the percentage of those who could not access the service in relation to those who used it.

**Table 4.** Sources of health and social care and medical advice.

Service	Used the service	Could not access it	Unmet need
Neurologist/Paediatric Neurologist	84	4	4
GP	82	3	4
Physiotherapist	65	5	8
Occupational Therapist	52	5	9
Speech & Language Therapist	45	6	13
Specialist Ataxia Centre or clinic (London, Newcastle, Oxford, Sheffield)	41	19	46
Other Hospital Dr	39	4	10
Accident and Emergency (A&E)	31	1	2
Chiropodist or Podiatrist	29	8	28
Geneticist/Genetic Counselling	25	11	42
Counselling or psychotherapy	20	8	41
Paid carer/personal assistant	19	7	39
Social Services (social worker, meals on wheels, community transport etc.)	18	7	38
District Nurse visiting you at home	16	6	39

The data indicate percentages in relation to those PwA who responded to this question (n=347)

The most frequently used services were Neurologists and General Practitioners followed by Physiotherapy, Occupational Therapy and Speech and Language Therapy. These services appeared to be well provisioned, with less than 10% of respondents indicating they did not have the necessary access, with the exception of speech and language therapy with 13%. The other two services where no significant access problems were reported include medical provision from other disciplines (mostly cardiology, ophthalmology, ENT, rheumatology and orthopaedics from free text answers) and accident and emergency services which were used by more than a third of respondents. On the other hand, a considerable shortfall of provision is evident for the remaining services. In particular, nearly half of the respondents who wanted to attend one of the specialist ataxia centres were unable to do so. Genetic advice, counselling and home visits by district nurses, and provision from social services and care providers were also not accessible to around 40% of respondents.

Those who were able to access services provided further information on their experiences ( Table 5). The majority indicated that they were satisfied with the service (columns 1 & 2 Table 5), with an average of only 16% reporting that it did not meet their needs. The services where most issues were reported were, in order of magnitude, counselling, social services, district nurse and GP provision. In relation to positive feedback provided, it is noteworthy that just over one third of respondents indicated that the amount of provision was insufficient.

**Table 5.** Respondents’ rating of the services they used.

Service	Excellent service - met my needs	Good service - but need more of it	Poor service - doesn't meet my needs
Your GP (n=269)	39	38	23
Neurologist/Paediatric Neurologist (n=267)	44	43	13
Other Hospital Dr (n=126)	51	38	11
Specialist Ataxia Centre or clinic (London, Newcastle, Oxford, Sheffield) (n=132)	53	39	8
Geneticist/Genetic Counselling (n=80)	58	30	13
Accident and Emergency (A&E) (n=103)	50	30	20
Chiropodist or Podiatrist (n=92)	57	37	7
Physiotherapist (n=212)	39	46	15
Occupational Therapist (n=168)	43	48	8
Speech & Language Therapist (n=138)	49	38	13
District Nurse visiting you at home (n=52)	46	29	25
Counselling or psychotherapy (n=63)	30	40	30
Paid carer/personal assistant (n=61)	64	28	8
Social Services (social worker, meals on wheels, community transport etc.) (n=63)	27	44	29
**Average**	**46**	**38**	**16**

The data indicate percentages in relation to those PwA who responded to this question, numbers are provided for each service used. Please note that this does not match the number of respondents who used this service as presented in Table 6.

**Table 6.** Reasons for joining Ataxia UK.

Percentage of people ranking this as one of the top 3 reason	PwA	Friends & Family
To learn about ataxia causes, symptoms, research, treatments	75	78
Information about living with ataxia, ongoing	56	46
Supporting medical research with the aim of finding cures	40	48
Information about living with ataxia, around time of diagnosis	33	30
Helping you feel more confident to deal with your situation	26	26
Supporting Ataxia UK’s work to help people affected by ataxia	20	32
Connecting with other people affected by ataxia	19	8
Information about accessing State entitlements	16	12

The data indicate percentages in relation to the total respondents to this question (PwA: n=337 friends & family: n=50)

A final question related to what kind of support people with ataxia and their friends and relatives were seeking from Ataxia UK, the ataxia charity and support organisation ( Table 6). For both groups the most prominent reason was availability of information about the disease, i.e. ataxia causes, symptoms, ongoing research and available treatments. In addition, information about how to cope with ongoing symptoms was also sought. Just over 40% of people with ataxia and 50% of friends and relatives joined to support medical research that aimed to find a cure. Interestingly, less than a third of people with ataxia indicated joining in order to feel more confident about dealing with their ataxia or meeting other people with the condition.

## Discussion

This study aimed to investigate the medical and associated challenges people with a variety of ataxias have to deal with, how these impact them, as well as what sources of help they access to support them with their difficulties and their availability and success in meeting their needs.

### Medical challenges and their impact

The findings in relation to medical symptoms are broadly in keeping with the literature characterising the various ataxia types covered in this survey ^1, 3, 5, 25^. The only unexpected result was to find a particularly high prevalence of heart problems in the I-CA group as opposed to the FRDA respondents (as cardiomyopathy is a common symptom in FRDA). This might have been because the answer categories did not specify the type of heart problems, and the reported problems might have been more a reflection of age, with the I-CA group having an older age profile, rather than problems directly linked with the ataxia.

Overall, the results highlight the basic fact that ataxia impairs co-ordination and impacts on mobility, and most ataxias affect multiple systems beyond motor performance.

Even though the nature and frequency of the reported medical symptoms largely agreed with the literature, the additional question on which of these had the biggest impact on people’s lives produced some interesting results. Whilst many of the most frequent symptoms, such as problems with balance, walking or speech, also had the highest impact on the respondents, some others such as clumsiness and problems with standing, ranked at the bottom half of the impact list, with less than 15% of people with ataxia reporting these as one of their three most impactful symptoms. On the other hand, a few less frequent issues such as severe visual impairment and bowel incontinence were reported to affect respondents considerably. It should be noted that the high impact scores of the rarer symptoms are in part due to the way the current data were analysed, i.e. percentages were calculated on the basis of how many people had reported the problem rather than the total number of respondents. In the case of severe visual impairment, for example, only 21 of 266 respondents had reported the issue, and seven of these scored it as having major impact, reflecting 33% of this group. If these seven respondents are considered in the light of the whole group, this result drops to only 2%. We would argue, however, that this misrepresents the impact this impairment has on those who experience it. In fact, these two divergent interpretations of the results highlight the need for clinicians to explore all facets of a patient’s symptom complex and investigate the impact each has on their lives rather than assuming that this equates to the prevalence of symptoms.

The data furthermore highlight how certain groups of ataxia appear to be affected differently by the same symptoms. The CA-U group, for example, was mainly impacted by balance and walking issues, with the remaining symptoms being impactful in less than 30% of respondents. This closely aligns with the Patient-Reported Outcome Measure of Ataxia ^16^, where half of the items of the short version relate consequences of these problems, reflecting the importance these have for this group of patients. A possible explanation for this finding is that CA-U may well consist of a high number of patients with immune ataxias where the primary involvement is with the cerebellar vermis, rather than the hemispheres. The FRDA respondents, on the other hand, showed a wider range of health issues that scored as highly impactful in more than 50% of their respondents and reported a lower impact of balance and walking difficulties than the other groups. The emerging group variations results could reflect differences in severity of the symptoms. The FAIS ^15^, for example, reflects the changing relevance of certain issue across the severity range in its subsets for early and more advanced stages of disability in people with FRDA. For instance, the FAIS-LESS includes items such as “tripping over or stumbling when walking” (lower limb function) or “feeling frustrated/annoyed/irritable” (mood), whereas the FAIS-MORE reflects the escalation of these issues by including items such as “falling over when walking” and “feeling depressed/angry/upset/tearful”. In addition, variations in symptom presentation such as manner of visual impairment, or possibly the age of onset of symptoms, i.e. length of time a respondent will have lived with the symptom, and thus the impact it has on people at different stages of their lives or how this affects their attitudes to their disability and their coping strategies. For example, despite the fact that FRDA is more disabling (with most patients being wheel-chair users in their adult life) more respondents with CA-U reported the high impact that balance problems have on them. This could be explained by the fact that FRDA is an early onset disease and thus patients with FRDA have lived with the reduced mobility and the impact of their balance problems from a young age. Patients with late onset ataxia have enjoyed the largest part of their lives without any disability. The new onset of disability is therefore more likely to impact on their lives. Similar results have been reported by Wilson *et al*. ^7^ who found that disease duration was positively associated with mental quality of life in people with FRDA.

One of the limitations of the survey was that respondents were not asked about the severity of their symptoms or their mobility status which would have allowed us to relate the reported impact of symptoms to the nature and severity of the impairments. The current data thus do not permit to make any judgement towards which of the above, or indeed other alternative explanations, resulted in the observed group differences. However, such questions should be the focus of future research in order to refine intervention to support each individual in such a way that impact of symptoms is minimised, either by addressing the symptom itself or enhancing the person’s coping strategies.

### Day-to-day challenges

Respondents were presented with a wide range of challenges that might present and impact on their lives. Some of these related directly to some of the medical symptoms presented in the previous section, others were effects arising from living with the disability more generally.

Reponses in this section fell largely into three groups, issues reported by more than 50% of all people with ataxia, those reported by between 45-50% and those reported by less than 30%. Issues relating directly to medical symptoms were reported in line with the frequency and impact of these issues, i.e mobility issues represented two of the top three issues - with “getting around outside their homes” in first place with around 80% and “getting around inside their homes” in third place affecting around 60% of all people with ataxia. Communication was an issue for just under 50% whereas coping with pain was less of an issue with less 30% of respondents mentioning this challenge. There thus appeared to be a relatively direct relationship between the prevalence of a medical symptoms and the degree of challenges arising from these.

Importantly, emotional challenges were highly prevalent. The lack of control over their lives stood out with more than 60% reporting this issue, whilst dealing with negative emotions, and loss of dignity were also prominent factors. In addition, a high number of respondents reported issues with activities of daily living, being accused of being drunk as well as obtaining a diagnosis. Even though it was one of the less frequently mentioned issues, around 30% of respondents also felt discriminated against. The breadth and prevalence of these issues signal that people with ataxia have to deal with a wide variety of challenges, not all of which are directly related to specific medical symptoms. This again supports the point made earlier that consultations with health professionals need to look beyond the immediate health symptoms and identify ways of how best to support people with ataxia.

Similar to the previous section, the respondents with FRDA presented a slightly different picture to some of the other groups. In particular, more people with FRDA indicated issues with moving around in their homes, communication, obtaining a diagnosis, coping with pain and feeling discriminated against. The report on communication problems ties in with the higher prevalence of dysarthria symptoms reported in section 1, as do the results for coping with pain.

***Impact.*** The consideration of the impact of the challenges as opposed to their prevalence again highlights some interesting issues. Whilst mobility outside the home and lack of control maintain their highest ranking positions, the order of the remaining challenges has changed. Coping with pain, which was reported by less than 30% of respondents, has one of the highest impacts in over 50% of these. Communication problems are another issue that was not as frequent but is reported as high impact. On the other hand, feeling strong emotions and maintaining their dignity appear to not have as much impact as their prevalence would suggest.

Between group differences again show a variation in the degree to which different types of ataxia are impacted. Similar to the report on the medical symptoms, people with FRDA reported a higher prevalence but lower impact of communication problems. They also stand out as being the most impacted by pain. The results for this reflect the reports ^18^ where mobility, communication and pain issues were reported as part of the top 10 most impactful symptoms in their adults with FRDA, with mobility taking precedence and communication featuring at the bottom of that list. On the other hand, the group with CA-U stands out in terms of their difficulties in moving around their home, again highlighting the impact of mobility issues on this group ^16^. However, there was no consistency across other categories related to this issue, e.g. they did not differ from the other groups in terms of mobility outside the home, so this could represent an artefact in the data rather than a generalisable result.

### Other challenges

Further day-to-day challenges were explored in the next section, in this case friends and relatives were also asked to respond. Broad themes capturing these items divided into psychosocial issues such as depression, anxiety, lack of confidence, social withdrawal, as well as family/relationship breakdown, and financial and other support issues such as inability to work, accessing benefit/social services, adapted housing/transport. In addition, respondents were asked about their fatigue levels and how they managed their caring responsibilities.

Ranking the items by prevalence in all people with ataxia, problems with fatigue was highlighted as the most frequent issue by more than 70% of respondents. Psychosocial problems, such depression, low self-esteem, social withdrawal and loneliness, and worry about the future were also reported by more than 40% of this group. Between group comparisons repeated the previously observed patterns where people with FRDA reported some issues more than the other groups. Depression, loneliness and family/relationship breakdown stand out in particular, other issues include lack of occupation and suitable transport. This mirrors reports on related challenges in the previous section (feelings of a lack of control and strong emotions). This could again be due to the factor listed above, i.e. earlier onset of the disease might mean that social relationships were not as established as firmly as in other groups, leading to greater feelings of loneliness. In addition, many people with FRDA will never take on employment as the disease might already be too severe when they leave school, and a greater proportion of respondents might have been wheelchair users, leading to a higher prevalence of transport issues. Comparison to the literature is difficult for these challenges as few other studies have reported these issues to date. The Patient-Reported Outcome Measure of Ataxia ^16^ scale includes a section on “mental” issues that focuses on emotional and mental wellbeing, social interactions etc., highlighting that such issues were raised by their participants with CA. The FAIS ^15^ reflects similar issues for the FRDA population. No information on prevalence of particular challenges is available from these two studies though. In addition, whilst demonstrating the relative importance of the symptoms reported ^18^, do not go into the level of detail reported in the current survey, although the high impact of emotional issues, social role and activity limitations might capture some of the issues reported by the present respondents with FRDA.

Issues reported by the friends and relatives group largely followed a similar pattern to the people with ataxia, i.e. coping with fatigue and being worried about the future featured as the two most prominent challenges experienced. As expected, the issue of managing caring responsibilities was more prominent in this group and featured to similar degrees as coping with fatigue. Interestingly, more than a third of people with ataxia also highlighted issues with this, suggesting that they also consider themselves carers of others. The number of respondents who fell into the group who had ataxia themselves and also cared for someone else with ataxia was too small to account for these results. We presume that, instead, this referred to caring for children and/or elderly relatives. This is an issue that has not been well explored in the literature where people with ataxia are usually considered as those being cared for. The fact that a considerable number of our respondents take on a carer role highlights that such questions need to be asked by medical and social care personnel assessing their needs in order to provide full support for this important role.

Another revealing result from this survey is the fact that, in addition to carer specific issues such as “managing caring responsibilities”, friends and relatives generally experienced similar issues to people with ataxia. Whilst some of the answers related to external challenges invariably affecting both groups such as transport problems, accessing benefits, etc., issues impacting on their mental health and wellbeing such as depression and loneliness were also highly prominent. One particularly interesting finding was that more than 40% of friends and relatives deliberately avoided social interactions. Whilst this fact can be explained by the presence of communication problems and negative emotions on the side of the people with ataxia, the fact that friends and relatives do not usually suffer from speech problems could mean that additional pressures are contributing to this, such as not wanting to socialise without the person with ataxia. The validity of this assumption would have to be investigated with further qualitative research, but mirrors to some degree the “loss of freedom” theme described by Draper *et al*. ^19^, which emerged from their interviews with carers of people with fast progressing neurological disorders, such as motor neuron disease or multiple system atrophy. Our data thus suggest that, in view of the importance of social interactions for mental wellbeing, family dynamics would be important to discuss with health professionals in order to maximise quality of life for those living with people with ataxia without exacerbating their feelings of isolation further.

The importance of carer welfare was underlined by a recent study which found that the presence of a carer was one of the main determinants to self-perceived quality of life in patients with spinocerebellar ataxia ^11^. The current study represents the first to have investigated the views of both people with ataxia and their friends and relatives. However, questions were limited to those that applied to both groups and a wide number of issues remain unexplored in the carer group in terms of the mental and physical wellbeing. Judging from previous reports on other progressive neurological disorders such as multiple sclerosis, Parkinson’s disease and dementia, the severity of impairment of the cared for person, the health and age of the carer, the availability of support, and perceived uncertainty are contributing factors to perceived carer burden ^20– 23^. Whilst we have no or limited information on the first two items, problems accessing support and uncertainty were issues that were frequently highlighted by our respondents as well, with the latter featuring as the most prevalent issue they had to cope with.

***Impact.*** In contrast to the previous sections, the reports on the impact of the reported challenges largely followed the same patterns as the frequency they were reported with. The only issue that deviated from this pattern was the presence of financial difficulties, which although not that common, understandably has a high impact on people’s lives. In addition, whilst this was reported as an issue less frequently by friends and relatives than people with ataxia, the impact was substantially greater for this group. Other noteworthy findings were the fact that although the FRDA reported more issues with family breakdown, none of these respondents signalled this issue as having particularly high impact on them. Challenges that stood out for the friends and relatives were the need to have housing adapted to the needs of the person with ataxia and also the management of caring responsibilities and discussing issues related to ataxia. The latter highlight further the need to provide those close to people with ataxia not just with practical support, but also to facilitate relationships and conversations within the close social circle, as already discussed above.

### Support provided

Respondents indicated that they had used a wide range of services in the past, and at least for essential services such as GP, A&E and neurology appointments, very low numbers said that they had not been able to access these. Other common services such as physio, occupational, and speech and language therapy were also generally well provisioned. On the other hand, there was a considerable unmet need in relation to access to counselling, social services, home visits by district nurses and paid care provision, as well as ataxia specific services such as genetic counselling and specialist ataxia centres. The lack of access to counselling services and social services is concerning considering the high number of non-medical challenges identified in this survey that should be addressed via these pathways. In addition, the value of specialist ataxia clinics has been highlighted before ^27^ and also reflects the literature on other progressive conditions where specialist, multidisciplinary care was highlighted as being highly supportive to both patients and their carers ^28^. There is thus a clear rationale for providing more people with ataxia an opportunity to be seen by such specialists.

The data on satisfaction levels for those services that had been accessed highlights further concerns. Although the numbers of those who perceived the service received as not having met their needs is small with an average of 15% of all people with ataxia, adding those who were positive about the service but felt they needed more of it results in a total of around 50% of respondents not having their needs met fully. Overall, provision that was viewed as not having met people’s needs included mostly generic services such a GPs, A&E or social work rather than those where professionals have a higher level of experience with ataxia and how to manage it, such as neurologist and the various AHPs. Knowledge and understanding of rare disorders in health and social care professionals (HCPs), or the lack thereof was a key theme highlighted as problematic in the study of the lived experience of patients with progressive neurological problems and their carers ^19^. Our study thus continues to highlight the need for further education of HCPs on how to deal with such disorders to allow them to provide more effective support for people with ataxia, make appropriate decisions on which other disciplines need to be involved and enable access to specialist clinics when necessary.

Finally, in answer to the question why respondents had joined Ataxia UK, it was positive to see that many respondents were willing to support medical research. Mostly, however, they were looking for information on ataxia and advice on how to live with the condition. Whilst it is reassuring that the charity can fulfil this function, it begs the question how much information is available to non-members, as current Ataxia UK membership represents less than 50% of the number of people thought to have ataxia in the UK. Information on the website is of course available to non-members, however, it will be important that healthcare professionals continue to raise people’s awareness of this resource at the time of diagnosis and provide sufficient explanation of the disease and its possible consequences, sources of support etc. themselves.

One other noteworthy result is that the wish to meet other people with the same condition featured relatively low on the list of respondents when compared to some of the other options around getting ataxia-specific information. This may reflect that the primary need is ataxia specific information which is not available elsewhere. However, it is still surprising that it does not feature higher in respondents’ priorities and this stands in contrast to anecdotal reports from people with ataxia on how much sharing information has supported them. It is worth noting that there are a number of reasons why people with ataxia may not want to attend support groups, such as low confidence levels, fear of finding out how their disease might progress, etc. Healthcare professionals therefore need to ensure that all their patients’ needs are met and not assume that in all cases charity support will fulfil that part of support.

### Limitations and future directions

This survey provides a snapshot of the challenges that people with ataxia and their friends and relatives experience and has generated some interesting data that should be followed up with more systematic research studies. However, due to the retrospective nature of the analysis, the current study suffers from a number of limitations. First, whilst we believe that the survey covered a wide range of such issues, it was by no means comprehensive, and some prominent challenges affecting people with ataxia might have been omitted. Some of the response choices could also have been refined to prevent unexpected results such as the higher prevalence of heart problems in the I-CA group. In addition, although the survey captured the views of several hundred respondents this only represents around 3.5% of the population living with ataxia and an even smaller percentage of their friends and relatives. We therefore cannot be certain to what degree the views reported here are reflective of those of the larger population. Having said that, a number of the survey results align with other reports in the literature, which might indicate that our findings can be generalised to the wider population to at least some degree.

Other limitations of this work include the fact that no information about the severity of the symptoms was collected to put respondent’s perceptions in context. In addition, the CA-U group consists of a heterogeneous group of patients with ataxia of unknown cause that are likely to have diverse aetiologies and possibly also diverse responses in relation to this survey. As we observed with the FRDA group, different types of ataxia may have different responses to this survey and thus by including all these patients as CA-U it is possible that any issues specific to some of these subgroups of ataxias have been lost to the whole group. 

Our current results, as well as the identified limitations of the survey provide a basis for developing future research priorities involving larger participant numbers and/or more refined data collection methods. For example, it is important to focus on impact as well as prevalence of symptoms, investigate how this varies across different ataxia genotypes and to what degree these issues are being addresses by the care provided in order to ensure that each individual receives the best care for their particular needs. In addition, further research on the views and support needs of carers, friends and relatives is required, both to ensure their wellbeing, and in extension, that of the person with ataxia they care for. In addition to collecting views on symptoms and other challenges, it appears to be crucial to gather further information how best to support these in order to ensure effective service delivery tailored to the specific needs of each ataxia population. Such research might also extend to capturing the views of HCPs, such as the barriers HCPS experience when dealing with people with rare disorders, and evaluation of interventions such as HCP training. Further evaluation of specialist multidisciplinary hubs such as the ataxia clinics supported by Ataxia UK will also form the necessary evidence base to replicate this model beyond the UK and for other rare disorders. In addition, more research is necessary on the most appropriate timing of intervention, e.g. early intervention to maintain level of functioning for longer, or later intervention when symptoms are more impactful in order to provide support at the right time without overloading services. Further investigations of service delivery models that would allow HCPs to support more people within their limited resources, such as group interventions as well as emerging telehealth technologies to facilitate effective selfcare, are therefore urgently needed.

## Conclusion

This survey highlights the multifaceted nature of the challenges people with ataxia and their friends and relatives have to deal with on a day-to-day basis, and the complex care needs that ensue. Whilst no definitive conclusions can be drawn to what degree this survey represents the views of the wider population, it has highlighted a number of important areas that require further, more in-depth investigation. Until such time, the survey serves to raise awareness of these issues and the need for HCPs to explore these issues with their patients in detail and identify the most appropriate multidisciplinary team to address them in order to provide effective, person centred care to promote health and wellbeing of all affected by this disorder. The survey also provides some pointers to assist in designing intervention trials that are assessing meaningful aspects of patients’ lives by highlighting symptoms with highest impact.

## Data availability

### Underlying data

University of Strathclyde KnowledgeBase: Data for: "The symptom burden of people with progressive ataxia and their friends and relatives", https://doi.org/10.15129/6279c12a-22ed-4f5a-98c1-b4a3ab2bcecc ^24^.

This project contains the following underlying data:

- Anonymised_data_file_Ataxia_UK_survey_2016_final.xls

### Extended data

University of Strathclyde KnowledgeBase: Data for: "The symptom burden of people with progressive ataxia and their friends and relatives", https://doi.org/10.15129/6279c12a-22ed-4f5a-98c1-b4a3ab2bcecc ^24^.

This project contains the following underlying data:

- Ataxia_UK_2016_Survey_transcript.pdf (survey questions)

Data are available under the terms of the Creative Commons Attribution 4.0 International license (CC-BY 4.0).
