# Supplementary material for: Symptom burden of people with progressive ataxia, and its wider impact on their friends and relatives: a cross-sectional study
Source: AMRC Open Res. 2021 Nov 30;3:28. [Version 1] doi: 10.12688/amrcopenres.13036.1 (PMC11064976; doi:10.12688/amrcopenres.13036.1)
Supplement: Peer review history

# Reviewer report: Reviewer response for version 1

**DOI:** 10.21956/amrcopenres.14111.r26954

**Published:** 2022-05-09

**Authors:** Carolyn Baylor

**Affiliations:** 1 Department of Speech and Hearing Sciences, University of Washington, Seattle, WA, USA

Thank you for the opportunity to review this paper. This paper can make a meaningful contribution to the literature regarding the experiences and impacts of ataxia not only on people with the diagnosis, but also on family/caregivers.

 Introduction:

 Page 4 – this line: “On the other hand, less children reported…” replace ‘less’ with ‘fewer.’

 The introduction gives a nice overview of the span of ataxia types to help the reader understand the diversity of presentations. No notable concerns with introduction.

 Methods:

 The age range of participants included in this analysis needs to be identified. Given the introduction, the assumption may be that anyone regardless of age was included in the survey, and thus in this analysis. However, it is quite easy to imagine that the life impact and impact on day to day activities varies between children and adults, and the impact on family relationships would vary, just by nature of the different types of activities, responsibilities, and relationships that exist for children versus adults. It seems that if all ages were included, the analyses should perhaps separate participants by age in recognition of this. I think there is danger in confounding results if the impact on a parent of a young child is mixed together with the impact on a spouse, as an example. Please clarify in the methods what age group is used in this paper and how that is managed in the analysis.

 Ideally, this survey would have gone through some cognitive interviews or pilot testing to ensure the questions are indeed relevant and understandable by participants. Clearly the authors of this paper cannot control that as it appears they are doing a secondary analysis of data that they were not involved in collecting, but this may warrant mentioning as a potential limitation.

 Results

 Any indication why the response rate was fairly low? Again the authors of this paper cannot control that since they did not collect the data, but a good question…how representative are these data?

 In the “respondent profile” section, it would be helpful to have a more detailed report of demographic information – move the age information here. Include information about gender? What other demographic information is available that might help to contribute to understanding of barriers/resources people may experience such as rural/urban living or other demographic information?

 Table 2 presents the list of different types of ataxia such as all the numbered variants of SCA. It would be very helpful in the introduction to include a brief explanation that there are multiple variants (or provide the number of variants) usually labeled as SCA1 etc. A detailed explanation of each variant is not needed, but the unfamiliar reader may get to Table 2 and wonder why all the numbers and what they represent.

 I see in the age profile there were very few respondents under the age of 20. Returning to the age issue, if those individuals were age 18 and above and were the people with ataxia, I could easily see including them as they are more adults than children. If those respondents were younger children, it may be worth considering removing them from the analysis and focus specifically on adults – again because the life roles and responsibilities of adults vary from children. Similarly, among the family respondents, if a notable proportion of them are responding on their experiences as caregivers to young children with ataxia, that is a different perspective than caregivers to spouse or similar relationships. I appreciate the likely desire here for a comprehensive view, but there is some risk that folding everything together without sorting out some of these differences may make the results harder to interpret. I think an interesting analysis would be to analyze separately the respondent/family data of parents of youth/children and report that separately than the respondent/family data of adults – include both analyses in the paper but that might be very interesting and a clearer picture of the data.

 I am concerned about including the three people who “expressed an interest in ataxia.” That could be someone, such as a healthcare provider, who has a professional interest but is not themselves personally impacted by ataxia. I would suggest removing them from the analysis if this is all that is known about them.

 Table 3: Possibly clarify the heading with “Percentage of all people with ataxia and within major subtypes reporting….” Or something similar – just clarifying what the percentages within each column are based on.

 Figure 2 – are the percentages calculated separately for each ataxia sub-type? For example, for balance, over 80% of the people with CA-U reported that this was a high impact symptom for them, but under 60% of people with FRDA reported this symptom was among the high impact for them? If that is correct, could the table heading be adjusted to clarify what the percentages are based on (individual diagnoses not diagnoses combined) in this figure? Same issue with Figures 3 – 6.

 Figures 5 and 6 – it is a little unclear how something gets categorized as a ‘wider challenge’ versus an ‘impact of a wider challenge’ as, per the phrasing of many of these, it is unclear how these are distinguished. Was this a distinction in the original survey itself or created for the analysis?

 Page 13 – caution with the word ‘significant’ since this is a descriptive analysis. Perhaps a different word here?

 Page 13 for this line:” The services where most issues were reported were…,” What is meant by issues – is this the same as unmet needs?

 Table 4 – please clarify this line differentiating the columns of could not access and unmet needs with this line in the text: “The final column represents the presumed unmet need of each service, expressed as the percentage of those who could not access the service in relation to those who used it.’' I’m just struggling a bit to sort out the two columns of could not access versus unmet needs as defined in this line.

 Discussion

 The point is well-taken that prevalence does not equal impact and the need for a patient-centered approach to looking at impact. Is this contradicted on page 15 with this line: “There thus appeared to be a relatively direct relationship between the prevalence of a medical symptoms and the degree of challenges arising from these.”

 Page 15 – are new acronyms introduced: FAIS-LESS and FAIS-MORE – the ‘less’ and ‘more’ acronyms are not defined. AHPs? HCPs / HCPS…

 In the discussion, be very cautious about drifting into language and conclusions that cannot be supported by a descriptive analysis only. For example, a statement such as this on page 16: “people with FRDA reported a higher prevalence but lower impact of communication problems.” We do not know that there actually is a higher prevalence or lower impact in the comparisons here because the these differences were not evaluated with inferential statistical analyses. We can say that “more people reported XYZ than reported ABC…” We can say more people or fewer people reported things, but we cannot say that an impact is higher or lower, or that there is a relationship between any variables with descriptive statistics. Also be very careful with wording such as “between group comparisons” – that wording suggests an inferential test.

 I suggest condensing the discussion. There are important key points, but these get lost a bit in the length and detail.

Is the work clearly and accurately presented and does it cite the current literature?

Partly

If applicable, is the statistical analysis and its interpretation appropriate?

Yes

Are all the source data underlying the results available to ensure full reproducibility?

Yes

Is the study design appropriate and is the work technically sound?

Partly

Are the conclusions drawn adequately supported by the results?

Yes

Are sufficient details of methods and analysis provided to allow replication by others?

Partly

Reviewer Expertise:

speech-language pathology

I confirm that I have read this submission and believe that I have an appropriate level of expertise to confirm that it is of an acceptable scientific standard, however I have significant reservations, as outlined above.

# Response

**Published:** 2023-01-20

**Authors:** Anja Lowit

**Affiliations:** University of Strathclyde, UK

Introduction:

 Comment: Page 4 – this line: “On the other hand, less children reported…” replace ‘less’ with ‘fewer.’

 Response: We have made this change.

 Comment: The age range of participants included in this analysis needs to be identified. Given the introduction, the assumption may be that anyone regardless of age was included in the survey, and thus in this analysis. However, it is quite easy to imagine that the life impact and impact on day to day activities varies between children and adults, and the impact on family relationships would vary, just by nature of the different types of activities, responsibilities, and relationships that exist for children versus adults. It seems that if all ages were included, the analyses should perhaps separate participants by age in recognition of this. I think there is danger in confounding results if the impact on a parent of a young child is mixed together with the impact on a spouse, as an example. Please clarify in the methods what age group is used in this paper and how that is managed in the analysis.

 Response: The youngest respondent was 18, so all responses from people with ataxia relate to the impact to life as an adult. However, we don’t have information on the age of people that are cared for by friends/relative respondents. This has been added as a limitation of the study.

 Comment: Ideally, this survey would have gone through some cognitive interviews or pilot testing to ensure the questions are indeed relevant and understandable by participants. Clearly the authors of this paper cannot control that as it appears they are doing a secondary analysis of data that they were not involved in collecting, but this may warrant mentioning as a potential limitation.

 Response: We agree that a limitation of the study is that the survey could have been refined to avoid unexpected results. Including a pilot stage as a solution in future surveys has been added to the discussion. Results

 Comment: Any indication why the response rate was fairly low? Again the authors of this paper cannot control that since they did not collect the data, but a good question…how representative are these data?

 Response: Although this paper represents a small proportion of people living with ataxia in the UK, the results do align with other reports in the literature. However, the low response rate is recognised as a limitation in the discussion.

 Comment: In the “respondent profile” section, it would be helpful to have a more detailed report of demographic information – move the age information here. Include information about gender? What other demographic information is available that might help to contribute to understanding of barriers/resources people may experience such as rural/urban living or other demographic information?

 Response: Age is already reported in the respondent profile section after the description of respondent categories.  We have added information on gender, but unfortunately no other demographic data were collected.

 Comment: Table 2 presents the list of different types of ataxia such as all the numbered variants of SCA. It would be very helpful in the introduction to include a brief explanation that there are multiple variants (or provide the number of variants) usually labeled as SCA1 etc. A detailed explanation of each variant is not needed, but the unfamiliar reader may get to Table 2 and wonder why all the numbers and what they represent.

 Response: This has been added to the introduction

 Comment: I see in the age profile there were very few respondents under the age of 20. Returning to the age issue, if those individuals were age 18 and above and were the people with ataxia, I could easily see including them as they are more adults than children. If those respondents were younger children, it may be worth considering removing them from the analysis and focus specifically on adults – again because the life roles and responsibilities of adults vary from children.

 Response: The youngest respondent was 18 so as the reviewer suggests, we consider them to be adults. We have pooled the data for 18-20 year olds with the 20-29 category in the figure to avoid any misconception that children might have contributed to the survey. A new figure has been uploaded.

 Comment: Similarly, among the family respondents, if a notable proportion of them are responding on their experiences as caregivers to young children with ataxia, that is a different perspective than caregivers to spouse or similar relationships. I appreciate the likely desire here for a comprehensive view, but there is some risk that folding everything together without sorting out some of these differences may make the results harder to interpret. I think an interesting analysis would be to analyze separately the respondent/family data of parents of youth/children and report that separately than the respondent/family data of adults – include both analyses in the paper but that might be very interesting and a clearer picture of the data. Response: This would be interesting for a future study. However, unfortunately information on the relationship to the person with ataxia was not included in this survey. This has been added a limitation of the study.

 Comment: I am concerned about including the three people who “expressed an interest in ataxia.” That could be someone, such as a healthcare provider, who has a professional interest but is not themselves personally impacted by ataxia. I would suggest removing them from the analysis if this is all that is known about them.

 Response: We have checked the responses of these individuals. They only responded to the later sections of the survey that related to them, with the only question reported in this paper relating to the reasons for joining Ataxia UK. As all data reported here are calculated as a percentage of those responding, their inclusion in this group has no effect on the results of the other sections. We have added this information to the manuscript.

 Comment: Table 3: Possibly clarify the heading with “Percentage of all people with ataxia and within major subtypes reporting….” Or something similar – just clarifying what the percentages within each column are based on.

 Response: We have added a note with the legend of the table.  

 Comment: Figure 2 – are the percentages calculated separately for each ataxia sub-type? For example, for balance, over 80% of the people with CA-U reported that this was a high impact symptom for them, but under 60% of people with FRDA reported this symptom was among the high impact for them? If that is correct, could the table heading be adjusted to clarify what the percentages are based on (individual diagnoses not diagnoses combined) in this figure? Same issue with Figures 3 – 6.

 Response: We had provided that information in the text at the start but have now added it to each figure and table.

 Comment: Figures 5 and 6 – it is a little unclear how something gets categorized as a ‘wider challenge’ versus an ‘impact of a wider challenge’ as, per the phrasing of many of these, it is unclear how these are distinguished. Was this a distinction in the original survey itself or created for the analysis?

 Response: For each section we report the prevalence of the issue (i.e. how many people experience it), as well as how much impact these issues have on people. This is an important distinction as the data show that the two don’t always go hand in hand. We have tried to highlight the distinction more clearly in the text at the start of each section.

 Comment: Page 13 – caution with the word ‘significant’ since this is a descriptive analysis. Perhaps a different word here?

 Response: We have reviewed the document and changed the wording where appropriate

 Comment: Page 13 for this line:” The services where most issues were reported were…,” What is meant by issues – is this the same as unmet needs?

 Response: We have rephrased this sentence

 Comment: Table 4 – please clarify this line differentiating the columns of could not access and unmet needs with this line in the text: “The final column represents the presumed unmet need of each service, expressed as the percentage of those who could not access the service in relation to those who used it.’' I’m just struggling a bit to sort out the two columns of could not access versus unmet needs as defined in this line.

 Response: We have tried to clarify this by adding additional information in the text and also removing one of the columns as it reported the same data in a different format which would have caused the confusion  

 Discussion

 Comment: The point is well-taken that prevalence does not equal impact and the need for a patient-centered approach to looking at impact. Is this contradicted on page 15 with this line: “There thus appeared to be a relatively direct relationship between the prevalence of a medical symptoms and the degree of challenges arising from these.”

 Response: We have changed this sentence to “There thus appeared to be a relatively direct relationship between the prevalence of a medical symptoms and the prevalence of challenges arising from these.” This statement refers to the problems people have, rather than the impact of the problem.

 Comment: Page 15 – are new acronyms introduced: FAIS-LESS and FAIS-MORE – the ‘less’ and ‘more’ acronyms are not defined. AHPs? HCPs / HCPS…

 Response: LESS and MORE refer to the stage of the condition, this explanation has been added to the text. Allied health professionals have now been defined.

 Comment: In the discussion, be very cautious about drifting into language and conclusions that cannot be supported by a descriptive analysis only. For example, a statement such as this on page 16: “people with FRDA reported a higher prevalence but lower impact of communication problems.” We do not know that there actually is a higher prevalence or lower impact in the comparisons here because the these differences were not evaluated with inferential statistical analyses. We can say that “more people reported XYZ than reported ABC…” We can say more people or fewer people reported things, but we cannot say that an impact is higher or lower, or that there is a relationship between any variables with descriptive statistics. Also be very careful with wording such as “between group comparisons” – that wording suggests an inferential test.

 Response: We have reviewed the discussion and removed any ambiguous statements of this nature

 Comment: I suggest condensing the discussion. There are important key points, but these get lost a bit in the length and detail.

 Response: We have significantly shortened the discussion

# Reviewer report: Reviewer response for version 1

**DOI:** 10.21956/amrcopenres.14111.r26953

**Published:** 2022-04-13

**Authors:** Adam Vogel

**Affiliations:** 1 Centre for Neuroscience of Speech, University of Melbourne, Melbourne, Vic, Australia

Thank you for the opportunity to review this study. The article deals with a hugely important aspect of disease and makes a valuable contribution to the field.

 The survey was distributed to 4500 households (with potentially multiple respondents in each household) yet only 418 responses (9%). This low response rate may accurately represent attitudes of the population however it is considerably lower than what would be considered a representative sample.

 It would be interesting to see the impact of health problems in relation to disease severity or disease duration.

 Its not clear why tables 1 and 2 were split.

 In Figure 4, ‘obtaining a diagnosis’ was listed as a day to day challenge. It’s not clear how 30-50% of respondents continue to have this as an issue, despite many of them already having a diagnosis.

 At the bottom of each figure it would be helpful to highlight group size to give perspective to the percentages shown.

 It’s not clear how figs 5 and 6 differ enough to warrant separate images.

 Can the authors consider discussing these outcomes in the context of the ‘voice of the patient’ report https://www.curefa.org/pdf/news/FA-Voice-of-the-Patient.pdf

Is the work clearly and accurately presented and does it cite the current literature?

Yes

If applicable, is the statistical analysis and its interpretation appropriate?

Yes

Are all the source data underlying the results available to ensure full reproducibility?

Yes

Is the study design appropriate and is the work technically sound?

Yes

Are the conclusions drawn adequately supported by the results?

Yes

Are sufficient details of methods and analysis provided to allow replication by others?

Yes

Reviewer Expertise:

Ataxia and PROMS

I confirm that I have read this submission and believe that I have an appropriate level of expertise to confirm that it is of an acceptable scientific standard, however I have significant reservations, as outlined above.

# Response

**Published:** 2023-01-20

**Authors:** Anja Lowit

**Affiliations:** University of Strathclyde, UK

Comment: The survey was distributed to 4500 households (with potentially multiple respondents in each household) yet only 418 responses (9%). This low response rate may accurately represent attitudes of the population however it is considerably lower than what would be considered a representative sample.

 Response: Although this paper represents a small proportion of people living with ataxia in the UK, the results do align with other reports in the literature. However, the low response rate is recognised as a limitation in the discussion.  

 Comment: It would be interesting to see the impact of health problems in relation to disease severity or disease duration.

 Response: This would be interesting for a future study, but unfortunately this data was not collected by this survey. This has been added as a limitation of the study.  

 Comment: It’s not clear why tables 1 and 2 were split.

 Response: Table 2 gives a breakdown of the diagnoses within the I-CA group. This information can’t be included in Table 1 because these people are already counted in the I-CA category. We have changed the table title to make this clearer.

 Comment: In Figure 4, ‘obtaining a diagnosis’ was listed as a day to day challenge. It’s not clear how 30-50% of respondents continue to have this as an issue, despite many of them already having a diagnosis.

 Response: We assume this relates to past experiences of obtaining a diagnosis and have added this to the manuscript.

 Comment: At the bottom of each figure it would be helpful to highlight group size to give perspective to the percentages shown.

 Response: This has been added

 Comment: It’s not clear how figs 5 and 6 differ enough to warrant separate images.

 Response: Figure 5 shows the challenges experienced, whereas Figure 6 shows which of these challenges have the greatest impact on the person’s life. Although on this occasion the two sets of data are similar, on previous occasions there were some notable differences between the prevalence and impact of issues in previous questions. We therefore feel that is best to retain the two figures, both for consistency and to highlight the difference in pattern.

 Comment: Can the authors consider discussing these outcomes in the context of the ‘voice of the patient’ report https://www.curefa.org/pdf/news/FA-Voice-of-the-Patient.pdf

 Response: We thank the reviewer for highlighting this resource which we have now integrated into the discussion.

# Reviewer report: Reviewer response for version 1

**DOI:** 10.21956/amrcopenres.14111.r26926

**Published:** 2022-02-10

**Authors:** Rita Horvath

**Affiliations:** 1 Department of Clinical Neurosciences, University of Cambridge, Cambridge, UK

The paper by Lowit et al. reports on a cross-sectional study based on a survey to evaluate the  disease burden of people with progressive ataxia, and its wider impact on their friends and relatives. This study investigates a very important aspect of ataxias, and trying to find out what are the most important problems patients, their relatives and carers face. The survey was heavily based on the involvement of Ataxia UK, and their members. The views of 366 people with ataxia and 52 friends and relatives were evaluated and summarized in this paper. The analyses was performed for the entire group, as well as for the three groups: Friedreich’s ataxia, inherited ataxia excluding Friedreich’s ataxia, and cerebellar ataxia of unknown cause. The results highlighted some important gaps in the clinical management of ataxias, and  also clarified what are the most important issues people with ataxia are facing. These data are valuable to highlight some relevant problems which should be improved in the future. The view of the patients and their family members and carers are also useful to design future developments to improve the diagnostic journey and management of the patients. There is indication that with the expanding novel information in basic and clinical research, general neurologists and GPs have difficulties in keeping up with the new opportunities in ataxias. People with ataxia would like to visit highly specialised ataxia centres to get expert advise.  

1. The analyses was performed for three groups: Friedreich’s ataxia, inherited ataxia excluding Friedreich’s ataxia, and cerebellar ataxia of unknown cause. While Friedreich`s ataxia is a genetically defined group, the other two groups are very heterogeneous. Interesting to see the large number of SCA6 patients in the responders, but very low number of SPG7 patients. If we consider that the frequency of SPG7-related ataxias is at least as high as SCA6, how can the authors explain this?
2. I think the Discussion is a bit too long and not well focused. It contains some repetition from the Results. The data are broken down in many categories and all three groups in each, which disturbs the flow. I think this could be shortened and focused to the main message.
3. I suggest to highlight in the Discussion that collecting and considering the opinion of patients and their relatives is extremely important to improve awareness of the problems, facilitates clinical research and leads to a higher involvement of patients in research studies and trials. 
4. I suggest to make a simple Table on the main results coming out of the study and show this may be divided in the subgroups and in total. Some of the current tables could go to a Supplement, if it is compatible with the journal.

Is the work clearly and accurately presented and does it cite the current literature?

Yes

If applicable, is the statistical analysis and its interpretation appropriate?

Yes

Are all the source data underlying the results available to ensure full reproducibility?

Yes

Is the study design appropriate and is the work technically sound?

Yes

Are the conclusions drawn adequately supported by the results?

Yes

Are sufficient details of methods and analysis provided to allow replication by others?

Yes

Reviewer Expertise:

neurogenetics, mitochondrial diseases, ataxias

I confirm that I have read this submission and believe that I have an appropriate level of expertise to confirm that it is of an acceptable scientific standard.

# Response

**Published:** 2023-01-20

**Authors:** Anja Lowit

**Affiliations:** University of Strathclyde, UK

Comment: The analyses was performed for three groups: Friedreich’s ataxia, inherited ataxia excluding Friedreich’s ataxia, and cerebellar ataxia of unknown cause. While Friedreich`s ataxia is a genetically defined group, the other two groups are very heterogeneous. Interesting to see the large number of SCA6 patients in the responders, but very low number of SPG7 patients. If we consider that the frequency of SPG7-related ataxias is at least as high as SCA6, how can the authors explain this?

 Reponse: This is representative of Ataxia UK’s database. Ataxia UK have many more members with SCA6 than SPG7. People with SPG7 might be more likely to join another support group.

 Comment: I think the Discussion is a bit too long and not well focused. It contains some repetition from the Results. The data are broken down in many categories and all three groups in each, which disturbs the flow. I think this could be shortened and focused to the main message.

 Reponse: We have significantly shortened the discussion and grouped the information differently to highlight the main message more clearly  

 Comment: I suggest to highlight in the Discussion that collecting and considering the opinion of patients and their relatives is extremely important to improve awareness of the problems, facilitates clinical research and leads to a higher involvement of patients in research studies and trials

 Reponse: Thank you for this suggestion, this has been added to the conclusion  

 Comment: I suggest to make a simple Table on the main results coming out of the study and show this may be divided in the subgroups and in total. Some of the current tables could go to a Supplement, if it is compatible with the journal.

 Reponse: We considered this suggestion however, due to the wide ranging data collected such a table would have been difficult to assemble in a clear manner, so we have retained the current structure.
